# Atomic Force Microscopy Reveals a Role for Endothelial Cell ICAM-1 Expression in Bladder Cancer Cell Adherence

**DOI:** 10.1371/journal.pone.0098034

**Published:** 2014-05-23

**Authors:** Valérie M. Laurent, Alain Duperray, Vinoth Sundar Rajan, Claude Verdier

**Affiliations:** 1 Univ. Grenoble Alpes, LIPHY, F-38000, Grenoble, France; 2 CNRS, LIPHY, F-38000, Grenoble, France; 3 INSERM, IAB, F-38000, Grenoble, France; 4 Univ. Grenoble Alpes, IAB, F-38000, Grenoble, France; 5 CHU de Grenoble, IAB, F-38000, Grenoble, France; University of Ottawa, Canada

## Abstract

Cancer metastasis is a complex process involving cell-cell interactions mediated by cell adhesive molecules. In this study we determine the adhesion strength between an endothelial cell monolayer and tumor cells of different metastatic potentials using Atomic Force Microscopy. We show that the rupture forces of receptor-ligand bonds increase with retraction speed and range between 20 and 70 pN. It is shown that the most invasive cell lines (T24, J82) form the strongest bonds with endothelial cells. Using ICAM-1 coated substrates and a monoclonal antibody specific for ICAM-1, we demonstrate that ICAM-1 serves as a key receptor on endothelial cells and that its interactions with ligands expressed by tumor cells are correlated with the rupture forces obtained with the most invasive cancer cells (T24, J82). For the less invasive cancer cells (RT112), endothelial ICAM-1 does not seem to play any role in the adhesion process. Moreover, a detailed analysis of the distribution of rupture forces suggests that ICAM-1 interacts preferentially with one ligand on T24 cancer cells and with two ligands on J82 cancer cells. Possible counter receptors for these interactions are CD43 and MUC1, two known ligands for ICAM-1 which are expressed by these cancer cells.

## Introduction

Adhesive interactions of cancer cells with the endothelium are key events in the metastasis process (i.e. the dispersion of cancer cells from one organ to other parts of the body) [1], [2]. During the formation and growth of tumors, cancer cells manage to escape from primary tumors and penetrate the blood flow, thus can travel over long distances. At distant sites within the human body, cancer cells interact with the endothelium, adhere and eventually extravasate, i.e. migrate through the endothelial barrier. Leukocytes and cancer cells use similar mechanisms for interacting with endothelial cells (ECs), but while the phenomena of adhesion and migration of leukocytes through the endothelium has been particularly studied during inflammation, few results are available regarding the role of the key molecules involved in the adhesion and transmigration of cancer cells [1], [3], [4], [5].

Similarly to leukocyte recruitment, tethering and rolling of tumor cells (TCs) on the endothelium have been demonstrated for some cancer cells and are mediated by selectins. After this initial interaction, firm adhesion takes place, mediated by several cell adhesion molecules belonging to the integrin family [6] as well as the Intercellular Adhesion Molecule-1 (ICAM-1) and Vascular Cell Adhesion Molecule-1 (VCAM-1) from the immunoglobulin family, leading to tumor invasion [7], [8]. VCAM-1 is expressed by the endothelium after stimulation, and interacts with the α4β1 integrin, while ICAM-1 is expressed by ECs, leukocytes and some TCs, and can be upregulated by inflammatory cytokines. ICAM-1 is involved in leukocyte adhesion to the endothelium through its interactions with LFA-1 and Mac-1 leukocyte integrins (β2 integrin). TCs lack β2 integrins, but neutrophils can act as a bridge between TCs and ECs, with LFA-1 on leukocytes binding to ICAM-1 expressed on both endothelial and TCs [5]. In addition, ICAM-1 is a receptor for other molecules, such as CD43 [9] and MUC1 [10], which are expressed by some TCs.

Cancer progression is associated with alterations in the expression of some adhesive molecules. Some works investigated the relationship between the N-cadherin expression and the progression of tumor malignancy [11], [12]. An increase of cancer cell invasiveness is combined with switching of E-cadherin by N-cadherin and an increase in the expression of some integrin sub-units [13]. From a quantitative point of view, the comparison of adhesive properties in non-malignant and malignant epithelial bladder cells have shown that an enhanced N-cadherin level in T24 malignant cells was accompanied by changes in unbinding properties of individual N-cadherin molecules [14]. In addition, the ICAM-1 expression has been associated with a more aggressive tumour phenotype [15], [16]. Nevertheless, the ligands involved in the firm adhesion of TC are not yet as clearly defined as for leukocytes, and the quantification of such adhesive interactions between ECs and cancer cells has not been investigated so far.

Quantitative information on the cell adhesive forces can be obtained using different force spectroscopy techniques: the bio-membrane force probe [17], optical tweezers [18] and the atomic force microscope (AFM) [19]. All these techniques operating under an optical microscope allow to visualise the cells and simultaneously measure adhesion forces from a few pN to a few hundreds pN or more. In this work, we choose to use the single-cell force spectroscopy mode of the AFM to study cell-cell interactions involved in the adhesion of TCs on ECs. In contrast with other methods of adhesion strength, this technique allows to carry out measurements in a configuration close to the *in vivo* situation. A cancer cell is attached to a soft cantilever and put in contact with an EC-monolayer and the force signal is monitored thanks to the AFM cantilever deflection [19], [20]. The signal also allows detecting events such as possible breakups of receptor-ligand bonds as well as the global adhesion strength at the cell level.

Determination of cell-cell interactions was carried out for different cantilever retraction speeds to study how rupture force (involved in cell-cell adhesive bonds) is modified. We investigated the relationship between the measured receptor-ligand bonds and the corresponding metastatic potential of human bladder cancer cells, in order to determine the adhesive signature of such cancer cells. Finally, we show that the ICAM-1 receptor on the ECs acts as a key mediator for the adhesive interaction with the most invasive cancer cells. Our findings indicate that the more invasive bladder cancer cells interact thanks to one or two types of ICAM-1 ligands: CD43 and MUC1 are good candidates, as demonstrated by flow cytometry experiments. This knowledge about such interactions is essential for the understanding of cancer cell adhesion to the endothelium, a mechanism leading to invasion and metastasis.

## Materials and Methods

### Cells and cell culture

Three bladder cell lines were used in this study: RT112, T24 and J82 (ATCC, Rockville, MD). These cell lines represent progression from well to poorly differentiated phenotypes and arise from superficial to invasive epithelial human bladder cancer. RT112 cancer cells are moderately differentiated and are characterized by a cytological grade 2 (or differentiation) [21]. T24 and J82 cancer cells are poorly differentiated and characterized by a cytological grade 3. To distinguish cancer cells from HUVECs, cancer cells were transfected with a plasmid expressing GFP (Green Fluorescent Protein – pEGFP). Human Vascular Umbilical Endothelial Cells (HUVECs) were purchased from Promocell (Heidelberg, Germany). Cancer cells were grown at 37°C in a humidified 5% CO_2_ atmosphere, in RPMI 1640 medium supplemented with 10% fetal calf serum, 100 UI/mL penicillin and 100 µg/mL streptomycin (complete RPMI medium). ECs were maintained in Promocell culture medium. The ECs were plated in complete culture medium on glass coverslips coated with collagen I (BD Biosciences, Le pont de Claix, France) and left 3 days to spread in order to achieve confluence. For AFM experiments, the culture medium was supplemented with HEPES (20 mM, pH 7.4).

### Atomic Force Microscopy

We used a Nanowizard II AFM (JPK Instruments, Berlin, Germany) mounted on a Zeiss microscope (Carl Zeiss, Jena, Germany). This configuration allows to carry out AFM measurements and simultaneously observe the cells using phase contrast or fluorescence modes. This AFM is also equipped with the 'CellHesion' module (JPK Instruments, Berlin, Germany). This module enables a long-range vertical displacement of the stage up to 100 µm which makes force spectroscopy measurements possible including cell-cell interactions. In parallel, a vertical piezotranslator (PIFOC, Physik Instrumente, Karlsruhe, Germany) is mounted on the microscope objective to move the objective concurrently with the microscope stage and focus on cells while carrying out AFM measurements. All the measurements were carried out at 37°C using the Petri Dish Heater (JPK Instruments, Berlin, Germany).

### Cantilever coating

Soft cantilevers were V-shaped ones without tips (MLCT-O, Bruker, France). They were calibrated using the thermal fluctuations analysis method [22] and exhibit a spring constant close to 0.01 N/m. To enable the adhesion of cancer cells to the cantilever, the latter was functionalized using biotin-conA (Interchim, Montluçon, France) [23]. After rinsing with PBS, cantilevers were incubated overnight at 37°C in biotin-BSA (Interchim, Montluçon, France) (0.5 mg/ml), rinsed again in PBS and then incubated in streptavidin (Interchim, Montluçon, France) (0.5 mg/ml) for 10 minutes. Finally, cantilevers were rinsed with PBS and set into a biotin-conA drop during 10 minutes then rinsed with PBS. This molecule allows binding of cancer cells to the cantilevers with a force larger than the cell-cell detachment force in our study: biotin-conA adheres to the cancer cell membrane with a detachment force of 2 nN [20], while the detachment force relevant in the interaction between cancer cell and EC is on the order of 1 nN.

### Cancer cell capture

**Figure 1:**
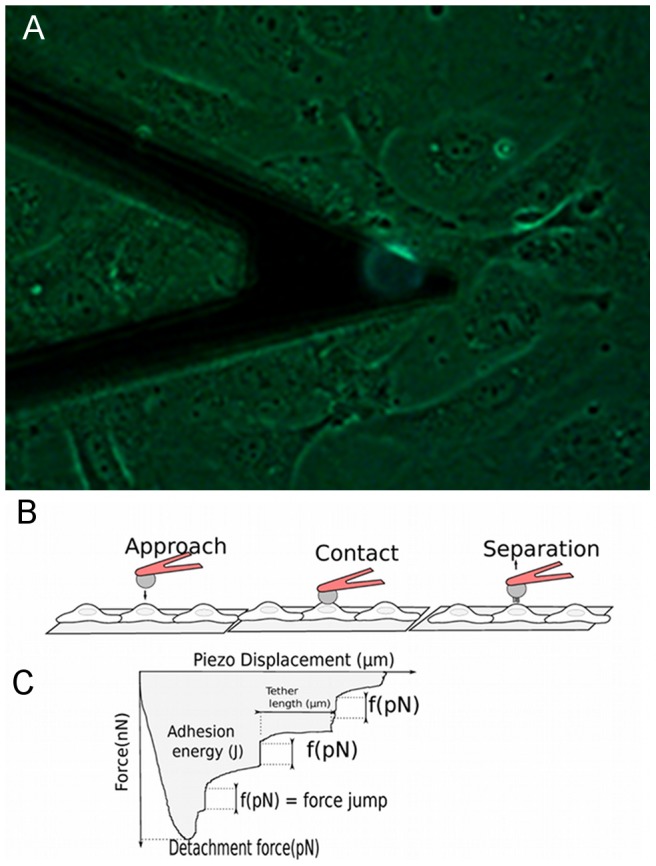
Interactions between cancer cells and ECs measured with AFM. A) Photograph of the cantilever with attached fluorescent cancer cell above the HUVEC monolayer. White scale bar corresponds to 20 µm. B) Sketch of the approach-retraction method and typical retraction force curve in terms of the piezo displacement. The cancer cell approaches the EC monolayer at constant speed. Then the cell comes into contact with the EC during 10 seconds (under 1 nN applied force) to create several bond complexes over the adhesion area. The cantilever is retracted at constant velocity in order to detach the adhesive bonds. The retraction curve shows force jumps corresponding to the rupture force (f) of bonds. The adhesive energy (shaded area) represents the detachment work done by the cantilever to completely detach the cell from the substrate. The detachment force is the force necessary to stretch the cancer cell and the EC until bonds start to detach. Note that some force jumps can follow a plateau corresponding to tether formation.

Cancer cells were grown in culture flasks, then were detached just before the AFM experiments, using a trypsin/EDTA solution (0.05% trypsin and 0.53 mM EDTA). RPMI medium with serum was added to the cells to block the effect of trypsin. Finally, cells were centrifuged and resuspended in medium without serum. Cancer cells were deposited in a Petri dish onto which an EC monolayer had been grown, and settled for a few seconds. Cell capture consisted in positioning the cantilever tip above a cancer cell (since cancer cells were fluorescent, they could be distinguished from ECs, see Figure 1), to come into contact with the cell during ten seconds with a force of 1 nN. Then the cantilever with the captured cell was retracted slowly at constant speed and the cell was kept in culture medium to rest for 15 minutes. Next, 1 ml of RPMI 1640 medium with serum was added. The cell was firmly bound to the cantilever and subsequently used to probe adhesion to ECs.

### Force spectroscopy: analysis of cancer cell-EC interaction

First, the cancer cell was set above an EC. The cantilever was lowered at constant low speed (1 µm/s) to put the cancer cell in contact with the EC (above the nucleus). A compression force of 1 nN was applied to the EC during 10 seconds (Figure 1A) in order to create bonds and to reproduce firm adhesion. Finally, the cancer cell was retracted with a speed ranging between 0.5 µm/s to 20 µm/s. Measurement of the cantilever deflection during vertical motion was recorded during the different stages (Figure 1B). Typically, for one cancer cell-EC pair, a sequence of five force curves was obtained at five different retraction speeds (with a rest time of about 1 minute between each curve). Then the cancer cell was left at rest during ten minutes and moved above another EC to measure a sequence of five force curves again. Finally, each cancer cell was used three or four times, therefore fifteen or twenty such force curves (N = 15 or N = 20) were obtained. The measurements were then collected for various cancer cell lines, during similar experiments. A sketch of the typical retraction force in terms of the piezo displacement is presented in Figure 1C. The minimum point on the curve is the detachment force, i.e. the force necessary to separate the cancer cell from the EC. The detachment force is supported by the cell deformability, but also by the number and strength of adhesive bonds formed between cells. The different jumps in force correspond to the successive breakups of bonds involved during cell-cell interaction [24], [25] and therefore represent rupture forces (i.e. receptor-ligand bonds). Note that a force jump can follow a plateau in force, corresponding to tether formation, whose extension is the plateau length. The retraction curve also provides information about the adhesion energy which is the work necessary to detach the cancer cell (shaded area in Figure 1C). This includes the work done to stretch the cells as well as the work done to break the molecular bonds [25]. All these parameters (detachment force, rupture force, adhesion energy) are obtained from the force curve using the Image Processing Software (JPK instrument, Berlin, Germany). For each set of conditions, AFM experiments were carried out about 9 times on 3 different days. Unless otherwise stated, data are reported as mean ± standard error of the mean. All statistical tests were performed using the R software (2.14 release). Since the data are correlated, we used a Generalized Linear Mixed Model (GLMM). Differences between the parameters calculated on untreated and anti-ICAM-1-treated cells were tested by the mixed function of the afex package in R software.

### Inhibition of ICAM-1 ligands on the ECs

Human monoclonal antibody to ICAM-1 [27] was used at a 30 µg/mL concentration. Before the AFM experiments, ECs were incubated for 15 minutes in the presence of the antibody at 37°C. Then cells were rinsed twice in PBS and incubated in 2 ml of culture medium.

### Immobilization of ICAM-1 and BSA

A 20 µL aliquot of recombinant ICAM-1 (RD Systems, Lille, France) (25 µg/ml) in 0.1 M NaHCO_3_ (pH 8.6) was adsorbed overnight at 4°C at the centre of the coverslip. Unbound proteins were removed by washing with PBS and 2 ml of complete RPMI 1640 medium were then added to the ICAM-1 coated dish before the AFM experiments.

For the BSA-coating protocol, a µL aliquot of BSA at 100 µg/ml in PBS was adsorbed 30 minutes at 37°C at the centre of the Petri dish. Unbound proteins were removed by washing with PBS and 2 ml of the complete RPMI medium were then added to the BSA coated dish before the AFM experiments.

### Flow cytometry analysis of ICAM-1, MUC1 and CD43 expression and immunofluorescence staining

Expression levels of ICAM-1 (on the EC surface), MUC1 and CD43 (on the cancer cell surface) were analyzed by flow cytometry (Accuri C6 flow cytometer, BD Bio-sciences). Quantification was made by measuring the geometric mean fluorescence. For immunofluorescence staining, glass coverslips were coated with 25 µg/ml human fibronectin. Cells were fixed with 2% paraformaldehyde, and processed for indirect immunofluorescence microscopy.

For measuring expression levels of ICAM-1 and MUC1, ECs or cancer cells were incubated with the primary antibody and then with FITC-conjugated (goat anti-mouse IgG) secondary antibody (Jackson ImmunoResearch, USA). The primary antibodies are a Human monoclonal antibody to ICAM-1 [26] or an anti-MUC1 monoclonal antibody C595 (Santa Cruz Biotechnology, Santa Cruz, USA). The anti-MUC1 antibody recognizes a tetrapeptide motif within the protein core of the MUC1 molecule.

For CD43 expression level, cancer cells were incubated with a monoclonal antibody CD43-clone L10 labeled with FITC (Invitrogen, USA), which reacts with the extracellular domain.

## Results

### Adhesion of different cancer cell lines

**Figure 2:**
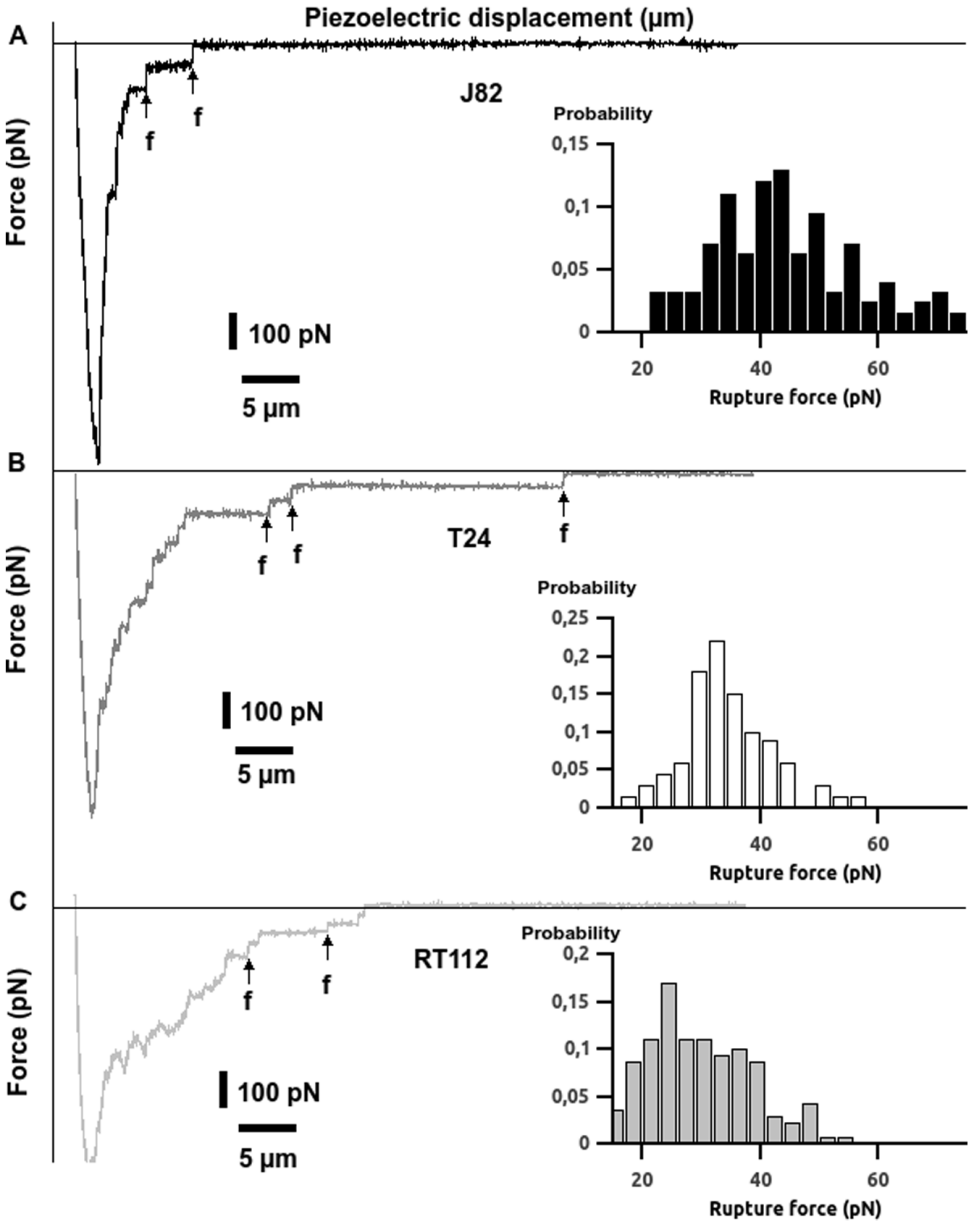
AFM force curves and rupture force histograms for different cancer cell lines. Typical force curves after 10s-contact between a TC and an EC on a HUVEC monolayer. Probability histograms with collected rupture forces f for J82 (A), T24 (B) and RT112 cells (C) at V = 5 µm/s. Vertical arrows denote examples of force jumps corresponding to breakup of receptor-ligand bonds.

To evaluate if cancer cell invasiveness is related to its adhesive properties, we carried out force spectroscopy measurements targeting the interaction between cancer cells (of different invasiveness) and ECs. The cancer cells arise from the following cell lines: RT112, T24 and J82. RT112 cells are the less invasive cells while T24 and J82 cells are the more invasive ones [21]. Force curves were performed between a cancer cell attached to the cantilever tip and a monolayer of ECs plated on a glass coverslip. Figure 2 shows typical force curves obtained during the interactions of the three cancer cell types (T24, J82 and RT112) with the ECs. Each retraction curve (retraction velocity V = 5 µm/s) shows several rupture events associated with the successive breaking of bonds involved during cell-cell interaction. Interestingly, the less invasive cells (RT112) present smaller rupture force steps as compared to most invasive cells (T24, J82). Moreover, the detachment force which is the minimum point (Figures 2A-B-C) of the curve is smaller for RT112 cells. Figures 2A-B-C also show the distribution of rupture forces detected for cancer cell interactions with ECs at V = 5 µm/s. These magnitudes [10–70 pN] are in the range of typical force values obtained for receptor-ligand bonds [23], [27], [28], [29]. It is noteworthy that the three cell types exhibit different force values: measurements reveal an average rupture force of 29.6±0.8 pN for RT112, 34.0±0.9 pN for T24 and 44.2±1.1 pN for J82 (V = 5 µm/s). Note that the average rupture forces are smaller for RT112 cells which are the less invasive kind.

### Adhesion energy and detachment force (cell level)

**Figure 3:**
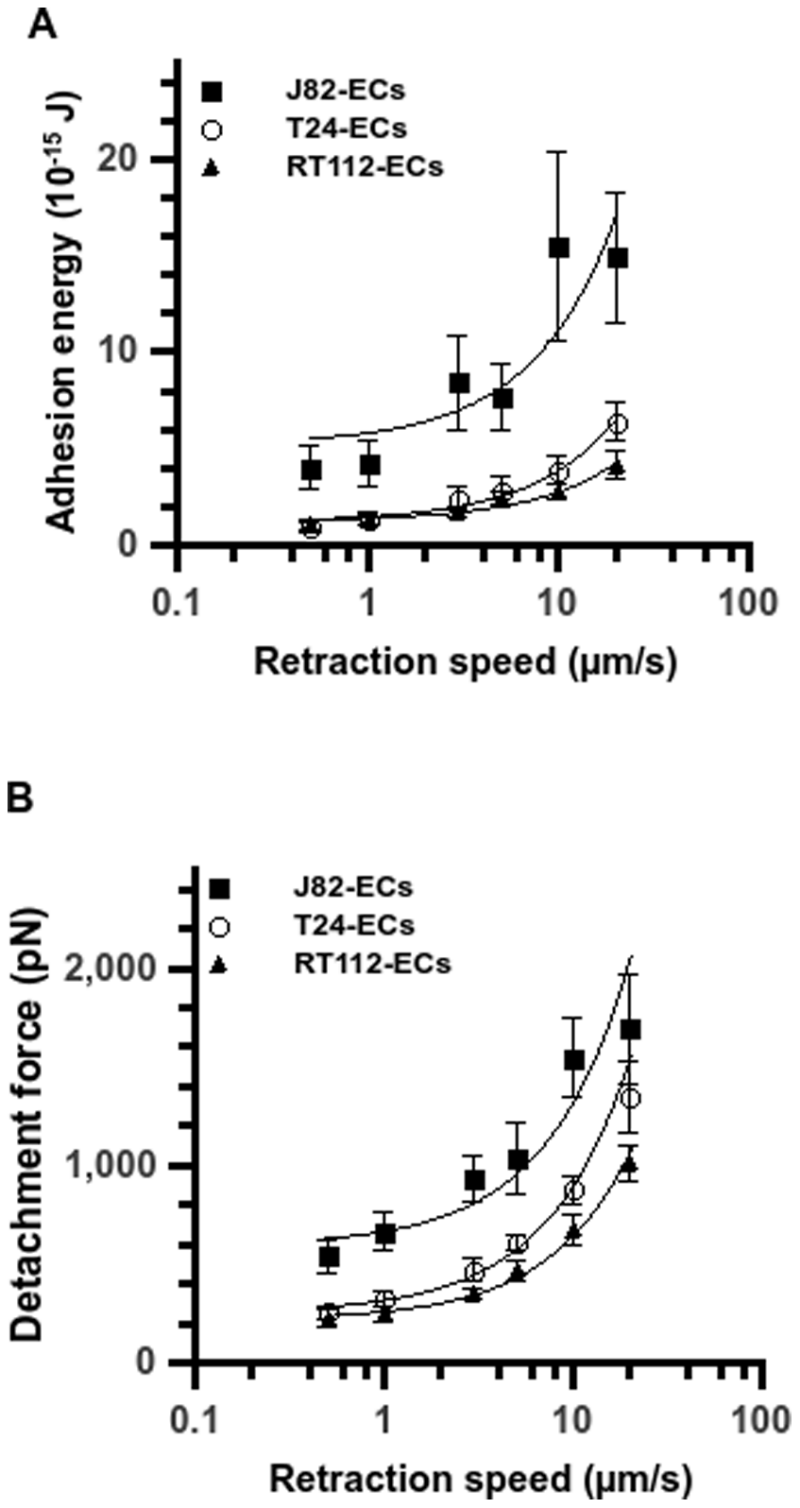
Adhesion energies and detachment forces for different cancer cell lines. Plot of the adhesion energy (A) and detachment force (B) vs. retraction speed after 10s-contact between a TC and an EC on a HUVEC monolayer. Three cancer cell lines: T24 (open circle), J82 (full square) and RT112 (full triangle). Data are plotted as mean ± standard error of the mean. The line is just a guide for the eye.

An important aspect to characterize the interaction of cancer cells to ECs is the adhesion energy which involves the whole cell contact area. The adhesion energy is derived through integration of the area below the curve F(z), where F is the force and z is the piezo displacement. The basis line is chosen as the final limiting value, after all bonds are detached. The JPK software allows to choose this value and then performs the integration. Therefore we investigated the adhesion energy as well as the detachment force (absolute value of the minimum force on retracting force curve, see Figure 1B) versus retraction speed (V). As shown in Figure 3, these two parameters increase with retraction speed. Regarding the adhesion energy, the most invasive J82 cells present larger values as compared to the T24 or RT112 cells. Moreover, this difference is confirmed by the detachment force values which are higher for J82 cells as compared to T24 and RT112 cells. In any case, detachment forces or adhesion energies are always smaller with the less invasive RT112 cell.

### Effect of retraction speed on rupture force

**Figure 4:**
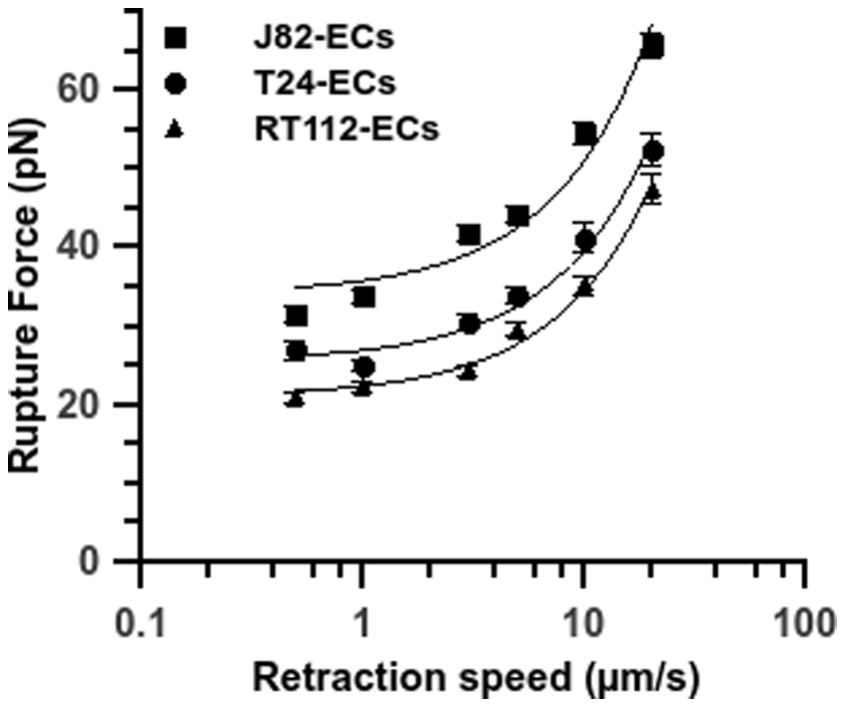
Rupture force vs. retraction velocity for different cancer cell lines. Relationship between rupture force and retraction speed after 10s-contact between a TC and an EC on a HUVEC monolayer. Three cancer cell lines: T24 (full circle), J82 (full square) and RT112 (full triangle) interacting with the endothelium. Data are plotted as mean ± standard error of the mean. The line is just a guide for the eye.

The rupture force has been shown theoretically to depend on the logarithm of the loading rate of the cantilever [30]. To study the signature of each cancer cell line, one needs to analyze the force spectra of the three cancer cell types during their interaction with ECs (Figure 4). These spectra present force values depending on the retraction speed or equivalently the loading rate r_f_ (N/s), equal to the product of the retraction speed V (m/s) times the spring constant k (N/m) of the cantilever, i.e. r_f_ = kV. Force values increase gradually with retraction speed and vary between 20.8±0.7 pN and 47.4±1.9 pN for RT112 cells, between 27.1±1.1 pN and 52.4±2.0 pN for T24 cells, and between 31.6±1.0 pN and 65.8±1.6 pN for J82 cells. For the three cancer cell lines, the average rupture force versus the logarithm of the retraction speed increases, but is not linear.

### Measurement of specific and non specific adhesion forces for cancer cells

**Figure 5:**
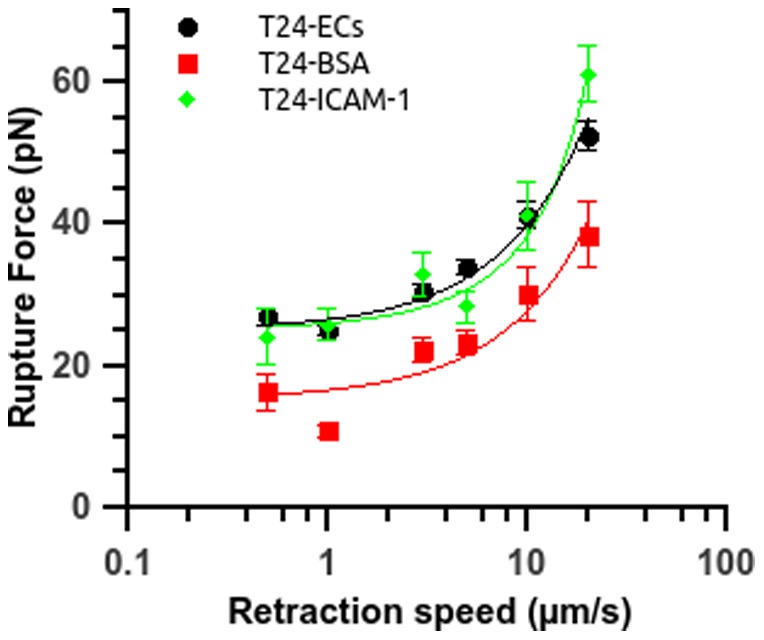
Control experiments for T24 cells interacting with recombinant ICAM-1 or BSA coated surfaces. Rupture force vs. retraction speed for T24 cells interacting either with a coated substrate or with ECs (circle). The substrate is coated with BSA 100 µg/ml (square) or recombinant ICAM-1 25 µg/ml (diamond). Data are plotted as mean ± standard error of the mean. The line is just a guide for the eye.

In a previous study, we showed that ICAM-1 was involved in TC extravasation [4]. To test the specific adhesion between cancer cells and ICAM-1 molecules, we carried out force spectroscopy experiments between a cancer cell attached to the tip of an AFM cantilever and immobilized ICAM-1 molecules on a glass dish. As shown in Figure 5, these measurements reveal that rupture forces are very close to the ones already obtained for cancer cell-EC interaction [28]. The values are in the range of [20–70pN] for a retraction speed between 0.5 µm/s and 20 µm/s. To compare these values to the non specific adhesion forces, we also measured the rupture forces between TCs and a BSA-coated surface. The force level involved in the non specific adhesion is in the range of [10–45pN] which is much less important, around 40% to 70% of the specific binding force.

### Role of the ICAM-1 receptor

**Figure 6:**
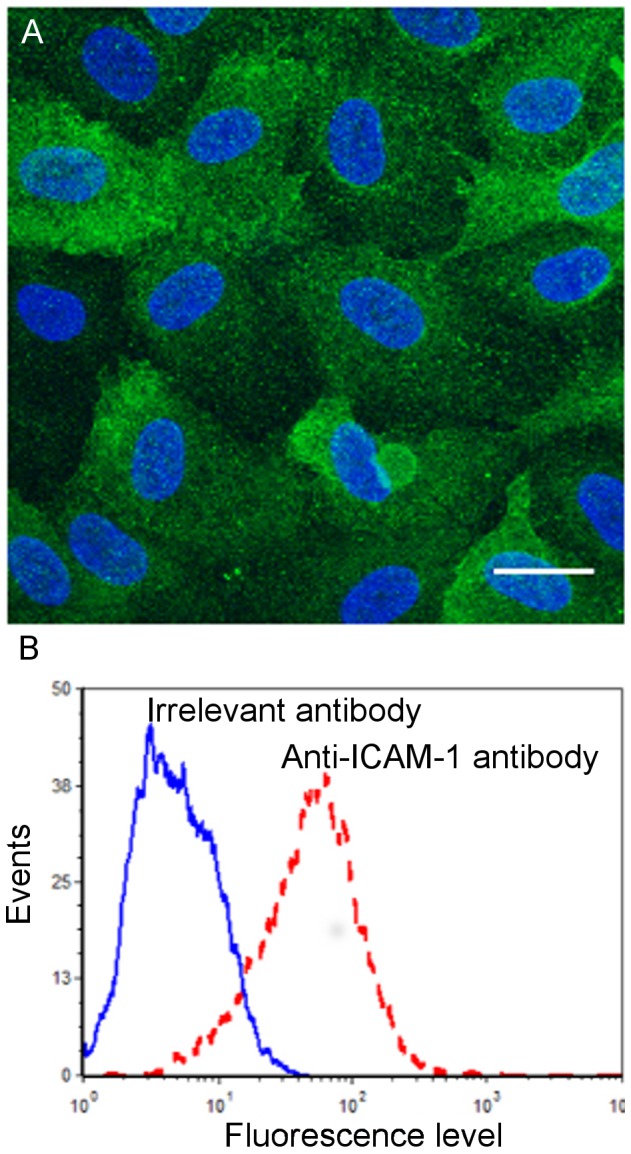
ICAM-1 expression on ECs. **A**) Confocal microscopy image of an EC monolayer stained for ICAM-1 (green). HUVECs were fixed with PFA. Nuclei are stained in blue using DAPI. **B**) Quantification of ICAM-1 levels by FACS analysis (dashed line) in comparison with an irrelevant antibody (solid line).

**Figure 7:**
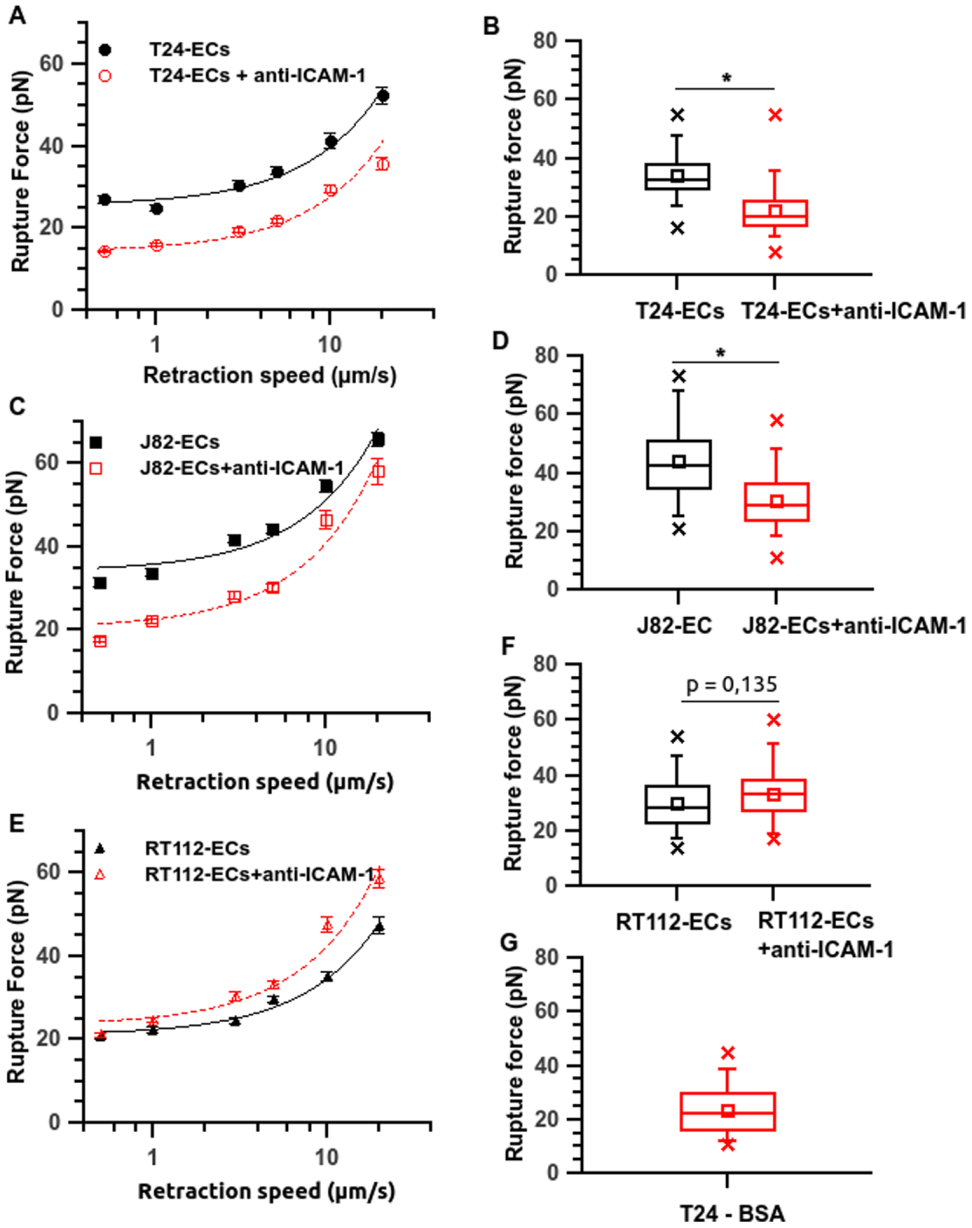
ICAM-1 is involved in the interaction between cancer cells and ECs. Rupture force vs. retraction speed after interaction between cancer cell and an EC, treated with an anti ICAM-1 antibody or not. Corresponding box-whisker plots show rupture forces at a retraction speed of 5 µm/s. (A, B) T24-EC, (C, D) J82-EC and (E, F) RT112-EC. As a comparison, the rupture force box plot is also shown for the T24-BSA interaction (panel G). For panels A, C and E, the line is just a guide for the eye. Data are plotted as mean ± standard error of the mean. Stars represent the p-value from GLMM statistical tests between parameters calculated on untreated and anti-ICAM-1-treated cells (*p≤0.05).

As shown by confocal microscopy imaging (Figure 6A), the expression of ICAM-1 on unstimulated ECs is moderate. FACS analysis (Figure 6B) confirms the ICAM-1 expression level, when comparing the fluorescence levels of cells treated with an irrelevant antibody and with the anti-ICAM-1 antibody. To determine the relative contribution of the ICAM-1 receptor on cell-cell adhesion, we examined the alteration of the adhesion forces when blocking this receptor with a specific monoclonal antibody. Figure 7 shows the effect of the antibody against ICAM-1 during the adhesion of the three cancer cell types with the ECs. Interestingly, inhibition of the EC ICAM-1 resulted in a significant decrease of the rupture forces for the more invasive cells only. For T24 cells, force averages varied between 27.1 pN and 52.4 pN (at different velocities) without blocking the ICAM-1 receptor, but between 14.4 pN and 35.7 pN, when blocking ICAM-1 (Figure 7A). This effect is also clearly visible on the box-whisker-plot obtained at a retraction speed of 5 µm/s (Figure 7 B). The anti-ICAM-1 antibody induces a significant decrease of the rupture force for T24 (from 34pN to 21.8 pN, see Figure 7B) and the value of 21.8 pN (+/−0.7) obtained after using of the antibody is comparable to the rupture force value of 23.3pN (+/−1.6) obtained for T24-BSA adhesion (see figure 7G). Therefore, after inhibition of ICAM-1, the rupture force level is typical of a non specific adhesion. This inhibition seems to be practically complete for T24 cells. For J82 cells, force averages vary between 31.6 pN and 65.8 pN without blocking ICAM-1 and between 17.7 pN and 58.1 pN when blocking ICAM-1. This effect of anti-ICAM-1 is clearly visible in the box-whisker plot of Figure 7D: the mean value decreases from 44.2 pN to 30.4 pN at a retraction speed of 5 µm/s. Finally, the adhesion of RT112 cells to ECs is not decreased in the presence of the anti-ICAM-1 antibody: the average values vary between 20.8 pN and 47.4 pN whereas they are between 21.1 pN and 58.6 pN when blocking ICAM-1. This non significant effect of the anti-ICAM-1 antibody is confirmed by the box-whisker plots (Figure 7F): the antibody does not induce any decrease in the rupture force. Therefore, an important reduction in binding forces for invasive cells (e.g. 35% for J82 and T24 cells) has been quantified here whatever the velocity, whereas there is no change in the case of the less invasive RT112 cell. This demonstrates clearly that ICAM-1 expressed by ECs plays a crucial role on the firm adhesion of the more invasive cells (J82 and T24).

### Detailed analysis of rupture forces

**Figure 8:**
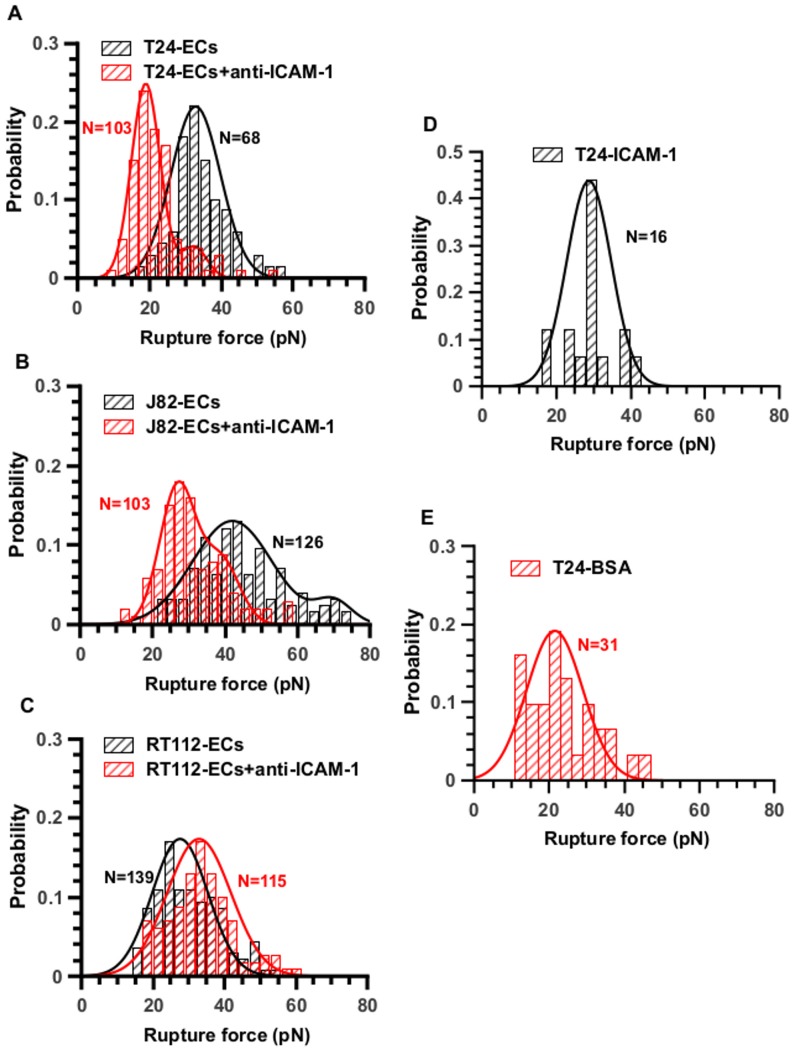
Distribution of rupture forces and effect of an anti-ICAM-1 antibody. Effect of an anti-ICAM-1 antibody on cancer-EC interactions. Rupture force distributions are Gaussian with one or two peaks revealing the presence of receptor/ligand bonds or non specific interactions. Probability histograms of rupture force (V = 5 µm/s) for (A) T24-HUVEC, (B) J82-HUVEC, (C) RT112-HUVEC. Black histograms represent interaction cancer-cell and EC without antibody whereas red ones show the force distribution after using the antibody. Panels D (T24-ICAM-1) and E (T24-BSA) show the rupture force probabilities for T24 cells in contact with coated substrates. The number N of events is indicated on the histograms.

Since we used average values of rupture forces which may hide the complexity of the adhesive bonds, a more detailed inspection of the force jumps (as shown using histograms) was carried out in order to gain more information. This analysis is given in Figure 8 where the TC-ECs or the TC-substrate (ICAM-1 or BSA) force jumps are recorded and presented using histograms, at a given velocity of 5 µm/s. Inspection of the histogram of T24-ECs rupture forces in Figure 8A (without the effect of anti-ICAM-1) reveals a Gaussian distribution centered at 32.9 pN (+/−5.8). Interestingly, this distribution of forces found for T24-EC interaction is quite similar to the one obtained during the interaction between T24 and the ICAM-1 coated-substrate (i.e. mean value  = 28.8 pN +/− 5.1) (see histogram in Figure 8D). On the other hand, when the ECs have been treated with the anti-ICAM-1 antibody, the peak in Figure 8A almost disappears (the area under the curve is divided by a factor of ten). A new peak centered at 19 pN appears, similar to the value of 21.5 pN found for T24 interacting with the BSA-coated surface (Figure 8E) which can be attributed to non-specific interactions.

The histogram of J82-EC rupture forces reveals a distribution of a double Gaussian distribution: there are two peaks initially (42 pN and 70 pN) as can be seen by the large spectrum of force values. After incubation with the antibody, the last peak (70 pN) completely disappears whereas the first one (42 pN) is lowered by a factor of 3 (Figure 8B). We may conclude that ICAM-1 is expected to interact with two ligands on the J82 cell surface, and that the antibody inhibits both interactions, but preferentially one. These bonds could be specific interactions with two different ligands for instance. In addition (Figure 8B), a new lower peak appears when using the antibody (≈28pN), whose value is very close to the one found for non-specific bonds.

The case of RT112 cell is different, as can be seen in Figure 8C. There is only one peak with and without the antibody located at similar levels (28 pN and 33 pN, no significant difference). The addition of anti-ICAM-1 antibody does not change the overall curve, therefore no clear effect is detected for RT112 cells, indicating that ICAM-1 is probably not involved in this adhesion process.

### Analysis of ICAM-1 ligands (CD43 and MUC1) expression by invasive cells T24 and J82

**Figure 9:**
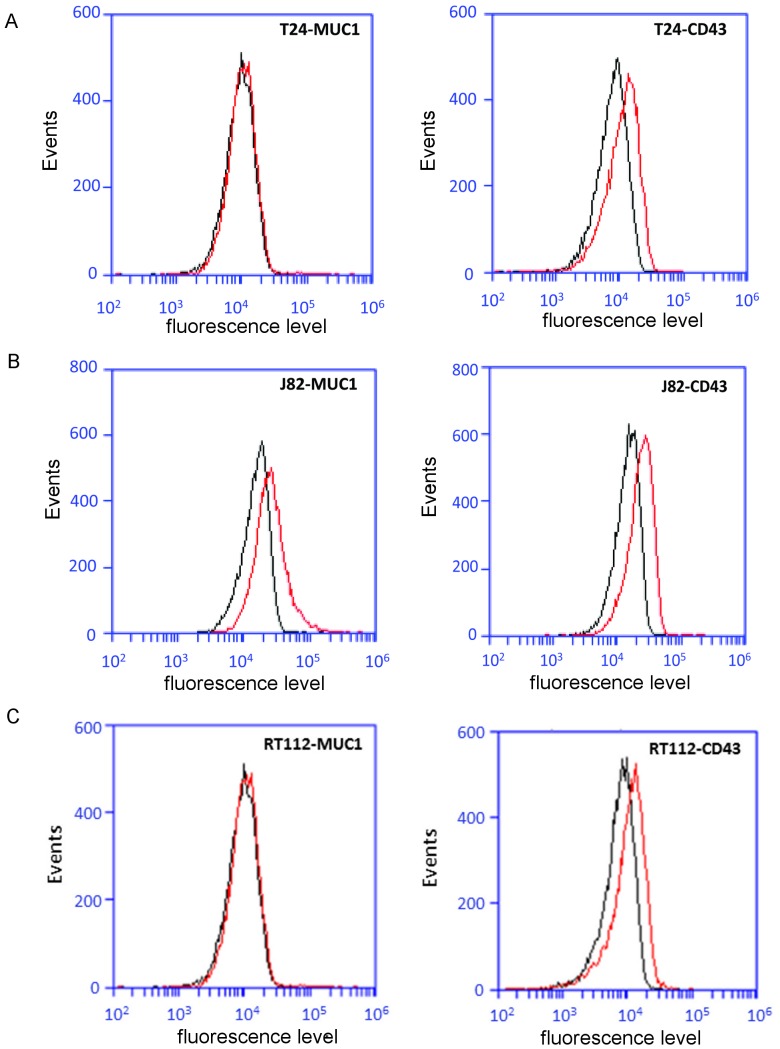
Expression of CD43 and MUC1 by the three bladder cell lines used in this study. Expression levels of CD43 and MUC1 (red line) by FACS analysis in comparison with an irrelevant antibody (black line): (A, D) T24 cells, (B, E) J82 cells and (C, F) RT112 cells.

Bladder cancer cells do not express the common ICAM-1 ligands, such as LFA-1 or Mac-1 [5]. On the other hand, the expression of MUC1 (Mucin 1) and CD43 (Leukosialin) were recently described as ICAM-1 ligands [31], [10], [9], [32]. Therefore, we quantified their expression by flow cytometry. The results in Figure 9 show that T24 cells express only the CD43 ligand while J82 cells express both CD 43 and MUC1 ligands. Concerning RT112 cells, they express only the CD43 ligand. These results are discussed below.

## Discussion

The mechanisms by which cells interact with the endothelium have been investigated in great details for leukocytes [20], [33]. Experiments using AFM have proven to be very useful for studying these interactions allowing to quantify the adhesive forces between a single leukocyte and an endothelial monolayer. Moreover, this technique helps to identify the molecules involved in the adhesion between cancer cells and the endothelium. Such experiments involving receptor-ligand bonds have been carried out by Zhang *et al.* [20], [25] using leukocytes in contact with ECs to investigate the role of VCAM-1, ICAM-1, selectins, β_1_ and β_3_ integrins. A qualitative study on cancer cell-EC interactions using AFM was also made by Puech *et al.* [24] but they showed no detailed analysis about these kinds of interactions. Therefore, this work focuses on the possible adhesion molecules and forces involved during interactions between bladder cancer cells (T24, J82 and RT112) and an endothelial monolayer.

The method used here is similar to previous works [19], [29], [24], since we catch cancer cells with the cantilever and lower it to make contact with the ECs. Then we retract the cantilever, to capture the signature of receptor-ligand bonds from the force signals, as shown schematically in Figure 1 and in real experiments (Figure 2). One question here is to determine whether such receptor-ligand bonds involve ICAM-1 when long tethers appear (Figure 1). Indeed Helenius et al. [28] showed that tethers usually correspond to the final part of the force curve, i.e. at long distances. Carpén et al. [34], on the other hand, showed possible connections of ICAM-1 to the cytoskeleton, suggesting the presence of bonds at shorter distances. In fact, this question still remains open as shown recently [35] since ICAM-1 can be found in filipodia, both on endothelial cells and on cells transfected with ICAM-1. This means that it is possible that tethers present ICAM-1 with or without any link with the cytoskeleton, and break far from the contact region, i.e. at large distances. Furthermore, it is possible that tethers may form on the cancer cell side. Finally, one may also consider the Jurkat cell-endothelial cell case [20] where the formation of long tethers and high adhesion energies is attributed to the presence of adhesion molecules such as ICAM-1, which are inhibited by antibodies (around 40% inhibition for the anti-ICAM-1 antibody). Thus it will be considered here that most receptor-ligand bonds arise from the presence of ICAM-1.

In our experiments, values of the adhesion energies are exhibited (Figure 3) for the two cell lines RT112 and T24, very similar to other studies [20], [25], [36]. On the other hand, J82 cells show higher values (≥4.0 10^−15^J) than the ones usually measured. This could be linked to the fact that they form two types of receptor-ligand bonds (Figures 8–9), as will be discussed below. In addition, the detachment force increases with the retraction speed and is larger for the more invasive cells. This result shows that adhesion at the cell level is higher for the more invasive cells and does not depend on retraction speed. As mentioned in the literature, we may assume that these higher detachment forces obtained with the more invasive cells in AFM stretching experiments are associated with higher cell stiffness [37]. Indeed, complementary elasticity measurements (data not shown) on the cell body using a spherical AFM probe confirm that the more invasive cells are more rigid (elastic modulus E = 493±138 Pa for J82 cells, 351±90 Pa for T24 cells and 246±95 Pa for RT112 cells). But, as was discussed before, the contribution of adhesive events (breaking of receptor-ligand bonds) also plays a role in the global cell response.

Different levels of rupture forces are obtained (Figure 4), ranging between 20 and 70 pN. These values are similar to those previously obtained in other studies [28] for different types of receptor-ligand bonds like cadherin-cadherin [38], [14], integrin-immunoglobulin [25], [39] or selectin-mediated bonds [40]. It clearly appears that the cells with the higher metastatic capacity show higher rupture force levels. This difference in adhesion strength for the three cancer cell lines studied is valid whatever the retraction speed [0.5–20 µm/s]. These data suggest that for different types of cancer cells, either various receptor-ligand pairs are involved or these pairs are regulated differently with more or less affinity [41].

To shed light into these mechanisms, we investigated the distribution of rupture forces at different retraction speeds (i.e. different loading rates), which exhibits multiple loading regimes, as seen by a continuous curve in the f-log(V) diagram (Figure 4). This behavior can be compared with the force-spectroscopy results obtained for leukocytes (expressing LFA-1) attached to substrates coated with ICAM-1 or ICAM-2 [42]. The meaning of the non-linear increase in rupture forces with retraction speed is related to the initial Bell's model [30] and its extensions by Evans and Richtie [43]. This theory predicts three different regimes of rupture force f vs. log(r_f_). In the first regime, f increases slowly; then at intermediate velocities, the force increases linearly vs log(r_f_) then we move into ultrafast regimes. Typically AFM data at classical velocities lie in the transition between the first two regimes where dependence is non-linear and increases monotonically. Only measurements on model systems (like streptavidin-biotin for example) exhibit a linear dependence. Usually with other receptor-ligand bonds, the response is always non-linear, and is more complex due to possible effects due to multiple types of bonds, leading to multiple barriers in the energy landscape [42]. Therefore the ICAM-1 receptor/ligand system presently characterized possibly involves multiple receptor-ligand types.

As shown by the values of the force jumps which are in the range [20pN–70pN], it appears that specific receptor-ligand bonds are present [28]. These interactions could involve endothelial adhesion molecules such as ICAM-1, VCAM-1 and E-selectin which have been shown to play an important role for the interactions between cancer cells and the endothelium monolayer [4], [7]. Previously, we identified the role of ICAM-1 for cancer cell adhesion to the endothelium [4]; therefore, we investigated here the role of ICAM-1 expressed on ECs on these adhesion mechanisms by using a specific anti-ICAM-1 antibody (Figure 6). The use of anti-ICAM-1 led to a significant reduction of the rupture forces for the more invasive cells (reduction of 35% on average for T24 and J82 cells) but no significant inhibition was found for the less invasive RT112 cells (Figure 7A to 7E). This result confirms the implication of ICAM-1 in the adherence of T24 and J82 cancer cells to ECs. The rupture force levels obtained for T24 cells when the ICAM-1 antibody is used (Figure 7A and 7B) are mostly in the range [15–40pN] for increasing retraction speed, a low value probably corresponding to non-specific bonds. This is indeed confirmed by force spectroscopy experiments with BSA-coated substrates, giving similar numbers (Figure 5 and Figure 7G). On the other hand, the levels of forces obtained between T24 cells and an ICAM-1 coated surface are identical (within experimental error) to the rupture forces measured during T24-ECs interactions, confirming the presence of an interaction with ICAM-1.

Finally, to understand the details of these interactions, we analysed the rupture force distributions at a given retraction speed (5 µm/s) through histograms and box-whisker plots as presented in Figure 8. First, this analysis is consistent with the previous results. The anti-ICAM-1 suppresses the specific interactions only for the more invasive cells: (i) the peak around 32.9 pN for T24 cells disappears when using the anti-ICAM-1 antibody (see Figure 8A) (ii) the high peak (70 pN) found for J82 cells completely disappears and the second one (42pN) is lowered by a factor 3 when using the antibody (see Figure 8B). Notably, the T24 cells and the J82 cells do not show the same number of peaks: this suggests that the ICAM-1 receptor on the EC interacts with one ligand on T24 cells and with 2 ligands on J82 cells.

Concerning the molecular structure linking cancer cells to the endothelium, since bladder cancer cells do not express common ICAM-1 ligands, such as LFA-1 or Mac-1, possible ligands on the cancer cell could very well be the MUC1 or CD43 ligands [31], [32], [44]. Geng *et al.* [44] demonstrated that efficient binding is possible between ICAM-1 and MUC1 whose expression increases with the invasivity of breast cancer cells [31]. Our measurements using flow cytometry demonstrated clearly that T24 cells express only MUC1 whereas J82 cells express MUC1 and CD43 (Figure 9). These results are in very close connection with the histogram analyses presented above, suggesting the presence of at least two ICAM-1 ligands on J82 cells and only one ligand on T24 cells. Note that for RT112 cells expressing only CD43, the endothelial ICAM-1 receptor does not seem to be involved, as shown by AFM spectroscopy.

To conclude, these experiments reveal the involvement of the endothelial ICAM-1 in the interactions between T24 and J82 TCs and ECs. The possible ICAM-1 ligands involved in this interaction are CD43 for T24 cells and both MUC1 and CD43 for J82 cells. Additional experiments are under way for studying the precise role of these two ICAM-1 ligands on these interactions. On the other hand, the less invasive RT112 cell does not adhere using this molecular scenario. This study provides a straightforward perspective for the understanding of the molecular mechanisms of the cancer cell-endothelium interactions. It leads to significant differences between cells of various metastatic potential and could provide interesting therapeutic means to block the extravasation process.
